# Supplementary material for: De-etiolation-induced protein 1 (DEIP1) mediates assembly of the cytochrome b6f complex in Arabidopsis
Source: Nat Commun. 2022 Jul 13;13:4045. doi: 10.1038/s41467-022-31758-7 (PMC9279372; doi:10.1038/s41467-022-31758-7)
Supplement: Supplementary file 1 — Supplementary Information [file 41467_2022_31758_MOESM1_ESM.pdf]

Supplementary Information

De-etiolation-induced protein 1 (DEIP1) mediates assembly of the cytochrome ***b*₆*f*** complex in Arabidopsis

Omar Sandoval-Ibáñez[1], David Rolo[1], Rabea Ghandour[1], Alexander P. Hertle[1], Tegan Armarego-Marriott[1], Arun Sampathkumar[1], Reimo Zoschke[1], Ralph Bock*[1]

[1] Max Planck Institute of Molecular Plant Physiology, Am Mühlenberg 1, 14476 Potsdam-Golm, Germany

* email: rbock@mpimp-golm.mpg.de

a

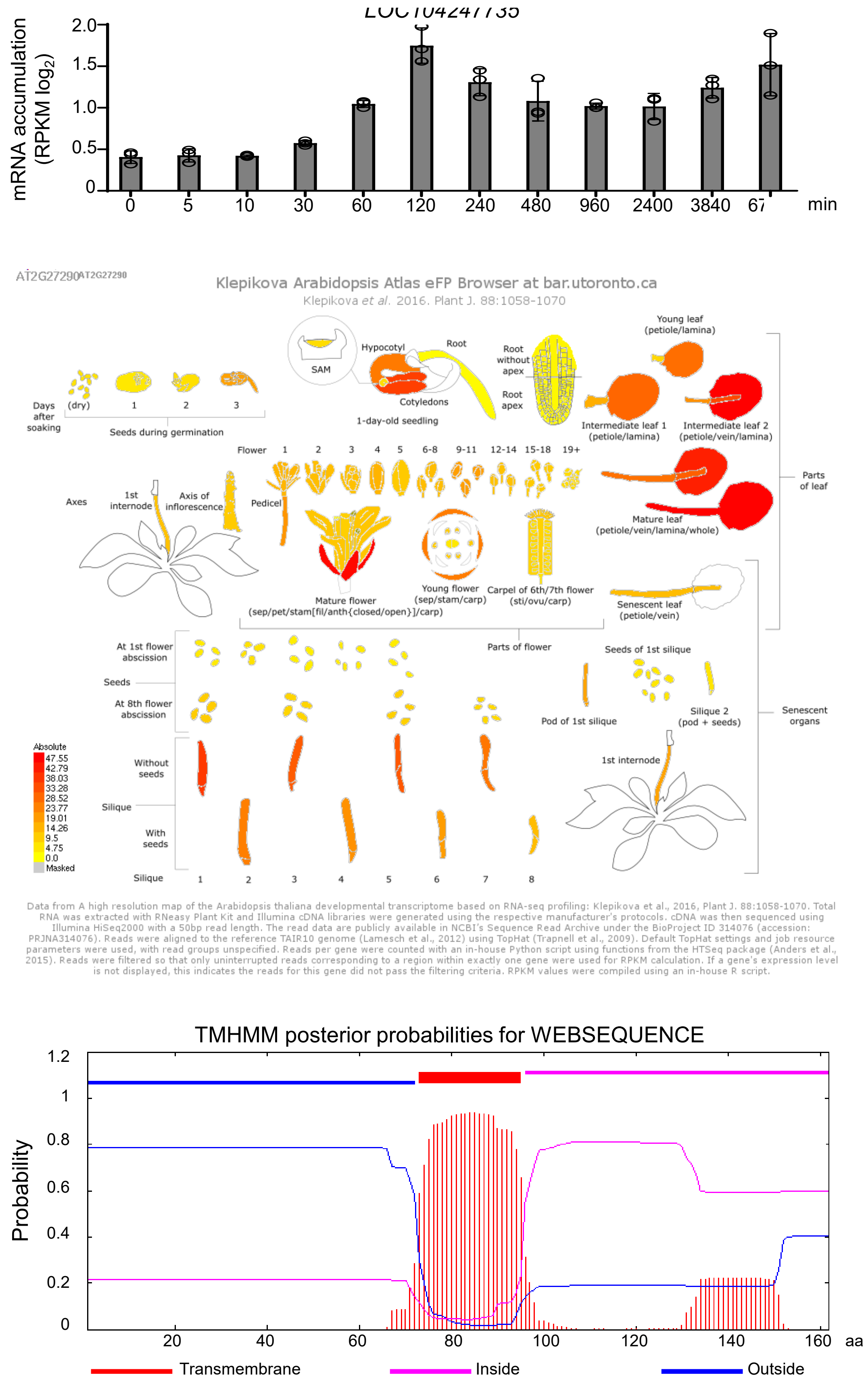
LOC104247735
mRNA accumulation (RPKM log_2)
2.0
1.5
1.0
0.5
0
0 5 10 30 60 120 240 480 960 2400 3840 67
min
AT2G27290
Klepikova Arabidopsis Atlas eFP Browser at bar.utoronto.ca
Klepikova et al. 2016. Plant J. 88:1058-1070
Days after soaking
(dry) 1 2 3
Seeds during germination
SAM
Hypocotyl
Root
Cotyledons
1-day-old seedling
Root without apex
Root apex
Young leaf (petiole/lamina)
Intermediate leaf 1 (petiole/lamina)
Intermediate leaf 2 (petiole/vein/lamina)
Parts of leaf
Flower 1 2 3 4 5 6-8 9-11 12-14 15-18 19+
Axes
1st internode
Axis of inflorescence
Pedicel
Mature flower (sep/pet/stam[fil/anth{closed/open}]/carp)
Young flower (sep/stam/carp)
Carpel of 6th/7th flower (sti/ovu/carp)
Mature leaf (petiole/vein/lamina/whole)
Senescent leaf (petiole/vein)
Parts of flower
At 1st flower abscission
Seeds
At 8th flower abscission
Seeds of 1st silique
Pod of 1st silique
Silique 2 (pod + seeds)
Senescent organs
1st internode
Absolute
47.55
42.79
38.03
33.28
28.52
23.77
19.01
14.26
9.5
4.75
0.0
Masked
Without seeds
Silique
With seeds
Silique 1 2 3 4 5 6 7 8
Data from A high resolution map of the Arabidopsis thaliana developmental transcriptome based on RNA-seq profiling: Klepikova et al., 2016, Plant J. 88:1058-1070. Total RNA was extracted with RNeasy Plant Kit and Illumina cDNA libraries were generated using the respective manufacturer's protocols. cDNA was then sequenced using Illumina HiSeq2000 with a 50bp read length. The read data are publicly available in NCBI's Sequence Read Archive under the BioProject ID 314076 (accession: PRJNA314076). Reads were aligned to the reference TAIR10 genome (Lamesch et al., 2012) using TopHat (Trapnell et al., 2009). Default TopHat settings and job resource parameters were used, with read groups unspecified. Reads per gene were counted with an in-house Python script using functions from the HTSeq package (Anders et al., 2015). Reads were filtered so that only uninterrupted reads corresponding to a region within exactly one gene were used for RPKM calculation. If a gene's expression level is not displayed, this indicates the reads for this gene did not pass the filtering criteria. RPKM values were compiled using an in-house R script.
TMHMM posterior probabilities for WEBSEQUENCE
Probability
1.2
1
0.8
0.6
0.4
0.2
0
20 40 60 80 100 120 140 160 aa
Transmembrane
Inside
Outside

Supplementary Fig. 1. Expression pattern of *DEIP1* in tobacco and Arabidopsis. a Expression of tobacco *DEIP1* in previously reported time-resolved de-etiolation experiments [39]. The x-axis gives the time of illumination from 0 (T0) to 6720 (T6720) minutes. The y-axis shows *DEIP1* transcript accumulation in RPKM. Error bars represent standard deviation; n=3 independent biological replicates. **b** Expression of *DEIP1* in Arabidopsis [41, 42]. The expression data for AT2G27290 were obtained from the eFP browser (bar.utoronto.ca). **c)** Presence of a membrane-spanning region in AT2G27290, as predicted by bioinformatic analysis [44]. The x-axis shows the amino acid position; the y-axis gives the probability of presence of a transmembrane segment.

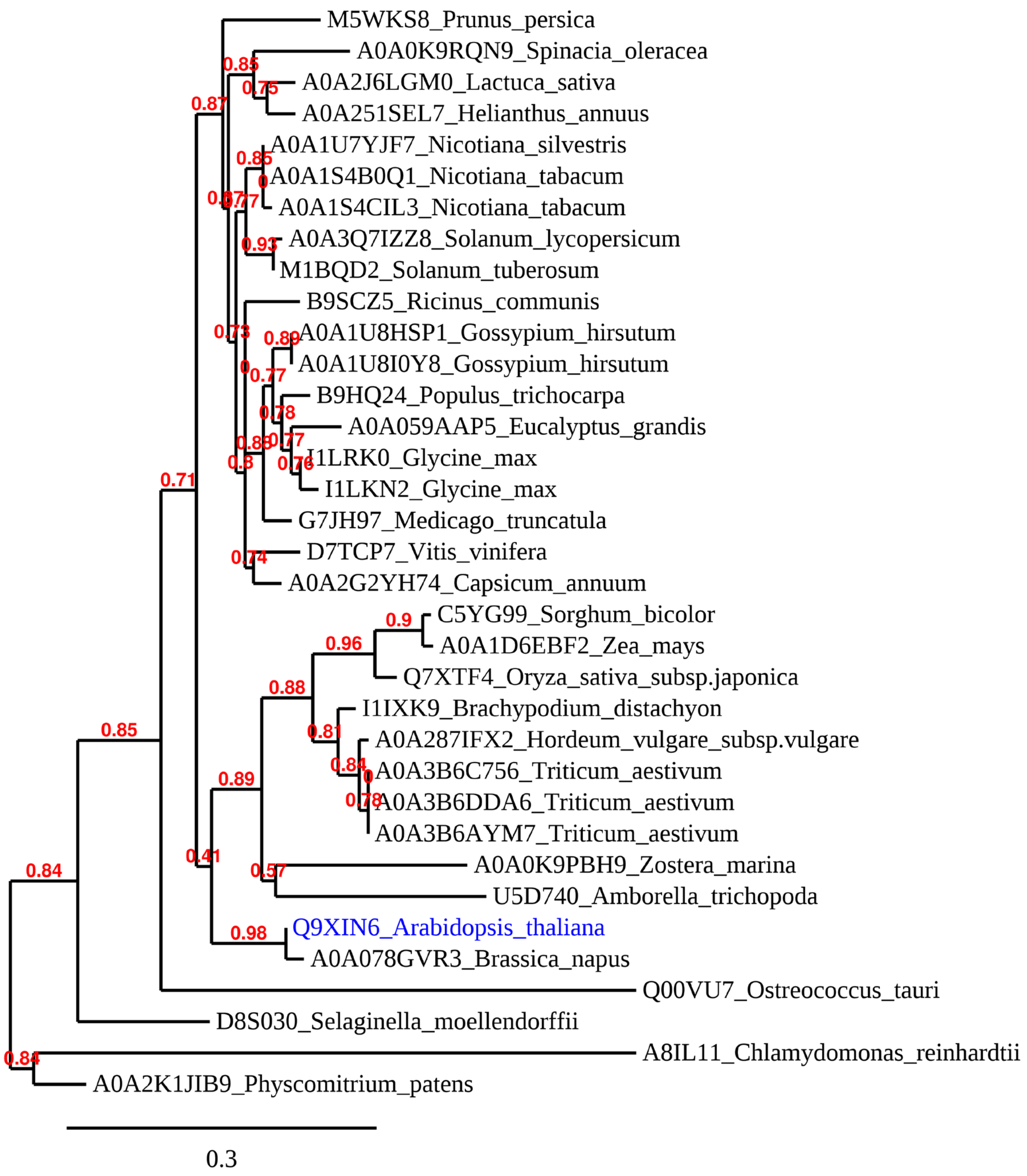

Supplementary Fig. 2. Phylogenetic tree of AT2G27290 (DEIP1). The phylogenetic tree was constructed with putative DEIP1 orthologs in plants and green algae. The UniProtKB protein entry and the species are given for each sequence. The protein corresponding to AT2G27290 is shown in blue. The red numbers indicate the branch support values. Detailed information on all proteins analyzed (including UniProtKB entry name, protein sequence, gene ID, protein name and protein length) can be found in Supplementary Table 1.

```
A8IL11_Chlamydomonas          -------------MQSTVQL----------TRRSTVVRRCEGRICRP-----------FA   26
A0A2K1JIB9_Physcomitrium      ----------MSLLSTDLVHEWRTSLPVHASPVVSSNVRPSSCGFRSKLKGGKVGPLSTM   50
C5YG99_Sorghum                ------------MAATALLLPSLAAAAPTPTQQLRLRLRRGSLACCS------------R   35
A0A1D6EBF2_Zea                ------------MAATALLLPSLAA----LTDTPRRAHR---LLQPQLQLQRGSLV--SR   39
Q7XTF4_Oryza                  ------------MAAGALLLANP------AAPTARHQHRLLQQRRQPRL---------LA   33
I1IXK9_Brachypodium           -----------MAAASALLLANLSA----PTGTARYRHR---LLQPRRQPPHQLLASRPL   42
A0A3B6C756_Triticum           ------------MAAPALPLANHAA----PTATARYLHRNHQWLQPK---RPGFLT--AR   39
Q9XIN6_Arabidopsis            ------------MAMMLLQI---------PPSSSLLNTRNLQIRFFH------------S   27
A0A078GVR3_Brassica           -------------MAMILQVPSSTP----SSLFHTRNTKTRFFFFSS-----------IQ   32
M5WKS8_Prunus                 -------------MATAVLVPCLN-----SASFFNNNGQSRNRCCAS-----------IQ   31
A0A1U7YJF7_Nicotiana_sil      -------------MASAWQI-----------CSQYYHNRIALCSISPTLPIFRKPNRVLN   36
A0A1S4CIL3_Nicotiana_tab      -------------MASASQISSH-----------YYHNRIALCSVSPALPILSKPNRVLN   36
A0A2G2YH74_Capsicum           ------------MMASALQV---------CSQYYYNNNRIAVCSLFTVFPNLRKPNKVLN   39
A0A3Q7IZZ8_Solanum_lyc        -------------MASALQI---------SSQY----SKIALCSLFPVFPNLRKPNKALN   34
M1BQD2_Solanum_tub            -------------MASALQI-------------SSQHSKIALCSLFPVFPNLRKPNKVLN   34
I1LKN2_Glycine                -------------MTTSLLLPSP------SCASILSKGTNRICTRAS-----------FH   30
G7JH97_Medicago               -------------MTTFSLLPSPS-----CASFLSNKKSSRFCSLAS-----------IQ   31

A8IL11_Chlamydomonas          PCH-PVSHI-----AKASPAT---EEPKTANKSEA--------ELATEKFGLEAGLLTAL   69
A0A2K1JIB9_Physcomitrium      PVLGLVGSKRSGVLCAATKEGKE-KEQDTKDIDA---------ETVTKKYGLEAGLWKIF   100
C5YG99_Sorghum                SVR-PPRRR---LAVSAVQETKD-GEAKTA-------------EEITEKYGLEFGLWKVF   78
A0A1D6EBF2_Zea                SLR-PPRRR--RLAVSAVQETKE-GEAKTA-------------DEITEKYGLEFGLWKVF   82
Q7XTF4_Oryza                  SSR-PPRWR-----LSAVQETKE-GEAQTA-------------EEITEKYGLEFGLWKVF   73
I1IXK9_Brachypodium           PLR-QPLRP-----LSAVQDTKE-ETAKTA-------------EEITEKYGLEVGLWKIF   82
A0A3B6C756_Triticum           PLR-QPRRA-----LSAVPETKE-EEAKTA-------------EEITEKYGLEVGLWKIF   79
Q9XIN6_Arabidopsis            SVS-ASSKK---FRCRAVREKA--EDIDKNISPPSSSPPPPSAEEVTKKYGLEVGLWKIL   81
A0A078GVR3_Brassica           TSS-ESNTN--KFRCRAVREKA--EEKNTSPPSP---------EEVTKKYGLEVGLWKIL   78
M5WKS8_Prunus                 PSKASSYSK--RFRVRALKEKTE-EEIKNPSSADSA-------EEITKKYGLEAGLWKIF   81
A0A1U7YJF7_Nicotiana_sil      SAT-SNTKI--KFKVRALKEKT--EEINSA-------------EEITKKYGLEVGLWKIF   78
A0A1S4CIL3_Nicotiana_tab      STT-SNSKN--KFKVKALKEKT--EEIKSA-------------EEITKKYGLEVGLWKIF   78
A0A2G2YH74_Capsicum           SAT-SNSNN--KFKVRALKEKTTEEEVKSA-------------EEITKKFGLEAGLWKIF   83
A0A3Q7IZZ8_Solanum_lyc        SVT-SNSNK--KFKIRALKEKTT-EEVKSA-------------EEITKKFGLEAGLWKIF   77
M1BQD2_Solanum_tub            SGT-SNSNK--KFKIRALKEKTT-EEVKSA-------------EEITKKFGLEAGLWKIF   77
I1LRK0_Glycine                SLK-SHVKK--GFRVRALKEKT--EEIESPSQPSSP-------EEVTKKYGLEAGLWQIF   78
G7JH97_Medicago               SRK-SNVKV---LRVRAVKEKT--EEIKSSSKQSSP-------EEVTKKYGLEAGLWKIF   78
                                              *                          :  * *:*** **

A8IL11_Chlamydomonas          TS--KDEGGE-------GKLSNTEQAKRLLAQYGSAYLITSISFAIVSFAACYLAVDSGV   120
A0A2K1JIB9_Physcomitrium      SS--KDKENS------PEQGTKTNQAKELLKRYGGAYLVTSISLSIVSFSLCYVLVQAGV   152
C5YG99_Sorghum                SS--KDDEEG----GGERKKSRTDQAKELLAKYGGAYLATSISLSIVSFTLCYLLISAGV   132
A0A1D6EBF2_Zea                SS--KEEEGG------EGKKSRTDQAKELLAKYGGAYLATSISLSIVSFTLCYLLVSAGV   134
Q7XTF4_Oryza                  SS--KEGEEE----EGKTRKSRTEQAKELLAKYGGAYLATSITLSLISFTLCYLLVSAGV   127
I1IXK9_Brachypodium           SS--KEEEEG----EGGKTKSRTDQAKELLAKYGGAYLATSITLSLISFTACYLLINAGV   136
A0A3B6C756_Triticum           SS--KEEEGE----EGKPKKSRTEQAKELLGKYGGAYLATSITLSLISFTACYLLINAGV   133
Q9XIN6_Arabidopsis            SS--KDDEGS--DGDNKKKKSKTDEAKELLAKYGGAYLATSITLSLISFSLCYVLVTSGV   137
A0A078GVR3_Brassica           TS--KDEESD--GETKKKKKSKTDEAKELLAKYGGAYLATSITLSLISFSLCYALVTSGV   134
M5WKS8_Prunus                 SS--KEEGKG-----GVENKSKGDDAKQLLAKYGGAYLATSIILSIISFSLCYALVSAGI   134
A0A1U7YJF7_Nicotiana_sil      SS--KEEGEE----ENKEKKSKGDQAKELLAKYGGAYLATSITLSLISFTLCYALINAGV   132
A0A1S4CIL3_Nicotiana_tab      SS--KEEGEE----ENKEKKSKGDQAKELLGKYGGAYLATSITLSLISFTLCYALINAGV   132
A0A2G2YH74_Capsicum           SS--KDEGDE----ENKDKKSKGDQAKELLAKYGGAYLATSITLSVISFALCYVLINAGV   137
A0A3Q7IZZ8_Solanum_lyc        SS--KEDRDE----ENKDKKSKGDQAKELLAKYGGAYLATSITLSLISFGLCYALINSGV   131
M1BQD2_Solanum_tub            SS--KEDGDE----ENKDKKSKGDQAKELLAKYGGAYLATSITLSLISFGLCYALINSGV   131
I1LKN2_Glycine                SS--KEEGKD----NSEQQKSKGDQAKELLAKYGGAYLATSITLSLISFALCYALISAGI   132
G7JH97_Medicago               SS--KEEGDQ--------QKSKGDQAKELLAKYGGAYLATSITLSLISFALCYVLINAGV   128
                               :  .                .   :* ** .**.*** *** :: :*:   *  : .*:

A8IL11_Chlamydomonas          DMAGVLARFGLEASDTSEKVGTFALAYAAHKALSPVRFPPTVALTPVVAKYLGKKKEEPS   180
A0A2K1JIB9_Physcomitrium      DVTSLLDKVGIHANDTGEKVGTFALAYAAHKALSPVRFPPTVALTPIVAGWFGKKPEDDN   262
C5YG99_Sorghum                DVQDLLAKVGIVTGETGGKVGTFALAYAAHKAASPIRFPPTVALTPVVASWIGKIRKGGD   192
A0A1D6EBF2_Zea                DVQDLLAKVGIVTGETGGKVGTFALAYAAHKAASPIRFPPTVALTPVVASWIGKIRKGGD   194
Q7XTF4_Oryza                  DVQDLLGKVGIATGETGGKVGTFALAYAAHKAASPIRFPPTVALTPVVASWIGRIKKGGD   187
I1IXK9_Brachypodium           DVQQLLTKVGIVTDETGGKVGTFALAYAAHKAASPIRFPPTVALTPVVANWIGKIRKGGD   196
A0A3B6C756_Triticum           DVQQLLGKIGIATDETGGKVGTFALAYAAHKAASPIRFPPTVALTPVVANWIGKITKGGD   193
Q9XIN6_Arabidopsis            DVQALLLKVGISTNETGEKVGAFALAYAAHKAASPIRFPPTVALTPIVANWIGKKVDKEK   197
A0A078GVR3_Brassica           DVQALLLKVGISTNETGEKVGAFALAYAAHKAASPIRFPPTVALTPIVANWIGKKVDKEK   194
M5WKS8_Prunus                 DVQALLQKVGISGGETGEKVGTFALAYAAHKAASPIRFPPTVALTPIVARWIGKKVEKEK   194
A0A1U7YJF7_Nicotiana_sil      DVQSLLQKVGISTDETGEKVGTFALAYAAHKAASPIRFPPTVALTPIVASWIGKKADKE-   191
A0A1S4CIL3_Nicotiana_tab      DVQSLLQKVGISTDETGEKVGTFALAYAAHKAASPIRFPPTVALTPIVASWIGKKADKEK   192
A0A2G2YH74_Capsicum           DVQALLQKVGISTDATGEKVGTFALAYAAHKAASPIRFPPTVALTPIVASWIGKKVDKDE   197
A0A3Q7IZZ8_Solanum_lyc        DVQSLLQKVGISTDETGEKVGTFALAYAAHKAASPIRFPPTVALTPIVATWIGKKVDKEK   191
M1BQD2_Solanum_tub            DVQSLLQKVGISTDETGEKVGTFALAYAAHKAASPIRFPPTVALTPIVASWIGKKVDKEK   191
I1LKN2_Glycine                DVQSLLQKVGISTDATGEKVGTFALAYAAHKAASPIRFPPTVALTPILAGWIGKKVEKDK   192
G7JH97_Medicago               DVQTLLQKVGISTDATGEKVGTFALAYAAHKAASPIRFPPTVALTPIVAGWIGKKADKDK   188
                              *:  :* ..*:    .. :.* :********* **:** *******::* :: .  .

A8IL11_Chlamydomonas          SGSNSK------   186
A0A2K1JIB9_Physcomitrium      DKTCS-------   267
C5YG99_Sorghum                ------------
A0A1D6EBF2_Zea                ------------
Q7XTF4_Oryza                  ------------
I1IXK9_Brachypodium           ------------
A0A3B6C756_Triticum           ------------
Q9XIN6_Arabidopsis            DDDK--------   201
A0A078GVR3_Brassica           DDE---------   197
M5WKS8_Prunus                 ------------
A0A1U7YJF7_Nicotiana_sil      ------------
A0A1S4CIL3_Nicotiana_tab      ------------
A0A2G2YH74_Capsicum           ------------
A0A3Q7IZZ8_Solanum_lyc        ------------
M1BQD2_Solanum_tub            ------------
I1LKN2_Glycine                ------------
G7JH97_Medicago               ------------
```

Supplementary Fig. 3. Sequence alignment of AT2G27290 (DEIP1) homologs from selected Viridiplantae species. An amino acid sequence alignment of AT2G27290 (DEIP1) and its homologs in plants and green algae is shown. The sequence corresponding to DEIP1 is highlighted in blue. The putative transit peptide (green letters), the domain of uncharacterized function 1279 (red) and the transmembrane segment (underlined and bold) of DEIP1 are marked in the DEIP1 sequence from *Arabidopsis*. Asterisks, colons and dots below the alignment mark amino acid residues that are fully conserved (*), conserved as amino acids with similar physicochemical properties (:), or partially conserved (.). The sequences are listed in Supplementary Table 1.

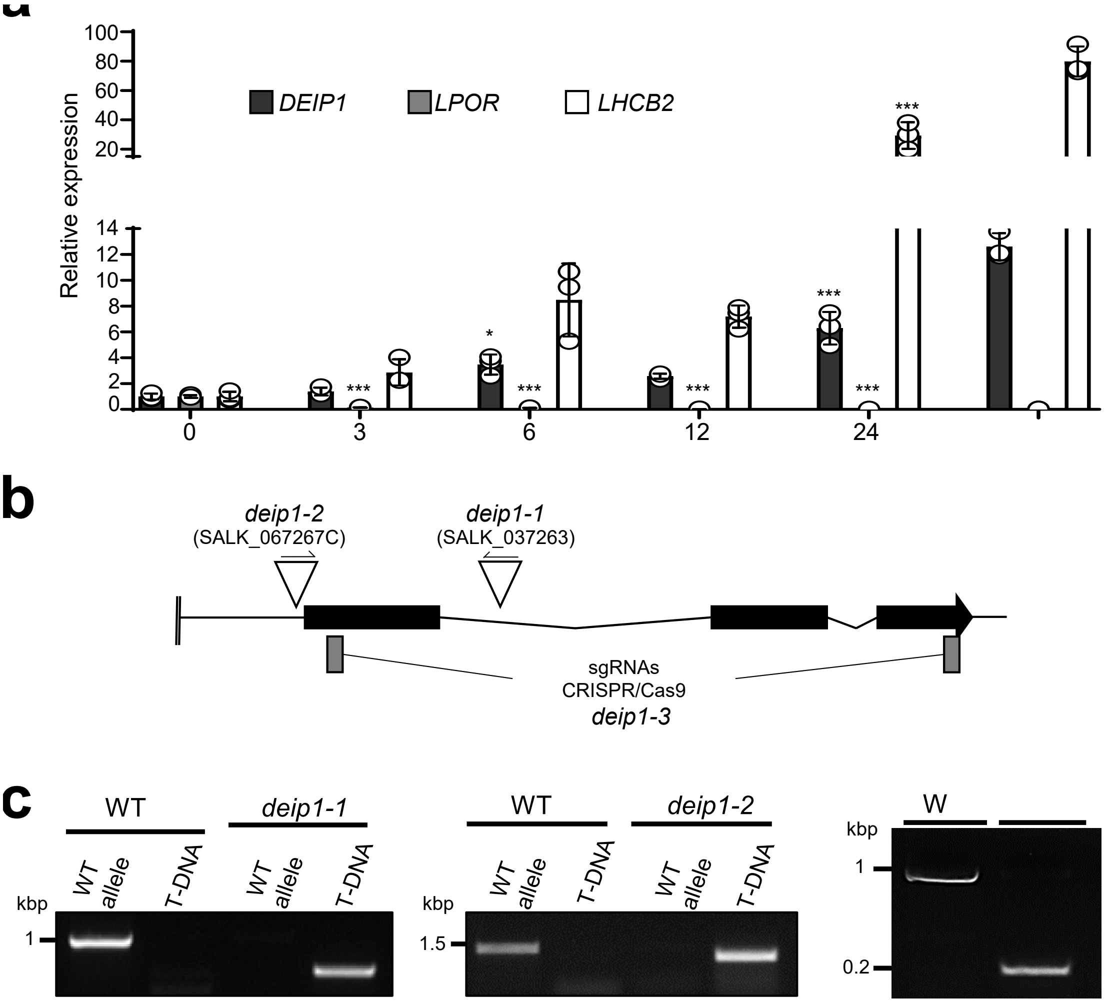

Supplementary Fig. 4. Transcript accumulation during de-etiolation of Arabidopsis seedlings, and isolation of *deip* mutants. a Time course of mRNA accumulation for *DEIP1*, *LPOR* and *LHCB2* in etiolated wild-type Arabidopsis seedlings upon illumination. mRNA accumulation was measured by qRT-PCR after etiolation (0 hours) and during de-etiolation for 3, 6, 12, 24 and 48 hours under continuous light. The data were normalized to *UBQ10*. n=3 independent biological replicates; error bars represent standard deviation; statistical significance was determined by one-sided ANOVA, *P* values were adjusted by Tukey post-test for multiple comparisons and compared to time point 0; * $P<0.05$, *** $P<0.001$. **b** Schematic map of the *DEIP1* locus, showing the location of the T-DNA insertions in mutants *deip1-1* and *deip1-2*, and the deletion induced by CRISPR/Cas9 genome editing in mutant *deip1-3*. **c** Genotyping of *deip1-1*, *deip1-2* and *deip1-3* mutants by PCR. For *deip1-1* and *deip1-2*, the left border of the T-DNA was used as target sequence for amplification of the mutant allele. For *deip1-3*, the full-length genomic sequence of *DEIP1* was amplified by PCR, and the product resulting from CRISPR/Cas9-mediated deletion is indicated by the asterisk. n= 3 independent biological replicates **d** Results of the sequence analysis conducted to characterize the deletion in the *deip1-3* mutant. The start codon (green), the deletion of 1085 bp (red) and the stop codon (blue) are indicated.

a

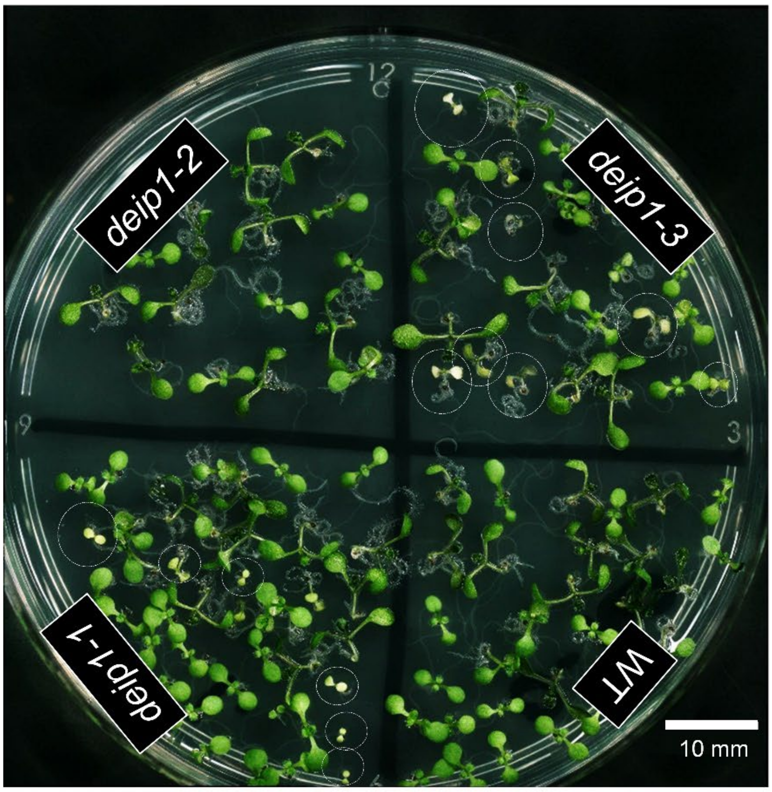

b

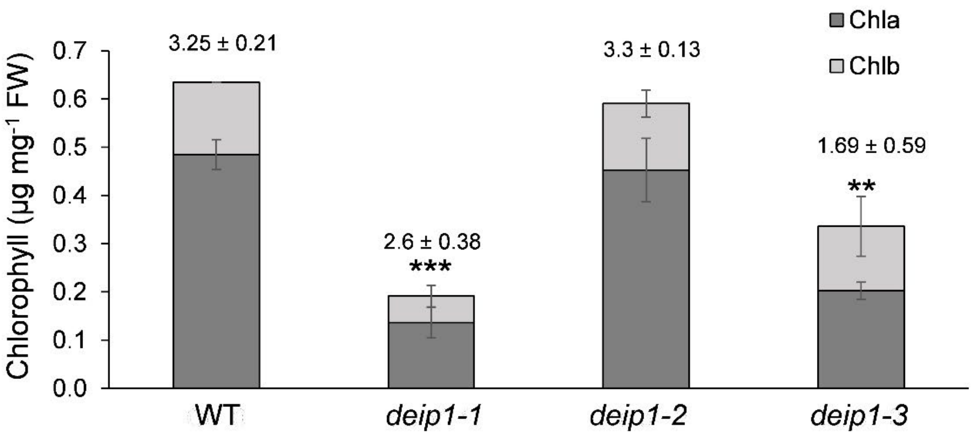

Supplementary Fig. 5. Seedling phenotypes and chlorophyll contents in wild-type plants and *deip1* mutants. a Phenotypes observed in seedlings from wild type (WT), *deip1-1*, *deip1-2* and *deip1-3*. The dashed circles mark pale plants observed in segregating progeny of heterozygous *deip1-1* and *deip1-3* mutants. n= 6 independent biological replicates. **b** Chlorophyll contents in 7-day-old seedlings of the wild type (WT) and homozygous *deip1-1*, *deip1-2* and *deip1-3* mutants. Chlorophyll was extracted with 80% acetone, quantified according to published protocols (see Methods), and normalized to fresh weight (FW). The contents of chlorophyll *a* and chlorophyll *b* are indicated as light grey and dark grey bars, respectively. The chlorophyll *a:b* ratio is shown above each bar. n=3; error bars represent standard deviation. Statistical significance was determined by one-sided ANOVA, *P* values were adjusted by Tukey post-test and compared to the wild type; ns, not significantly different in total chlorophyll content; asterisks represent significant differences in total chlorophyll content; ** $P<0.01$, *** $P<0.001$.

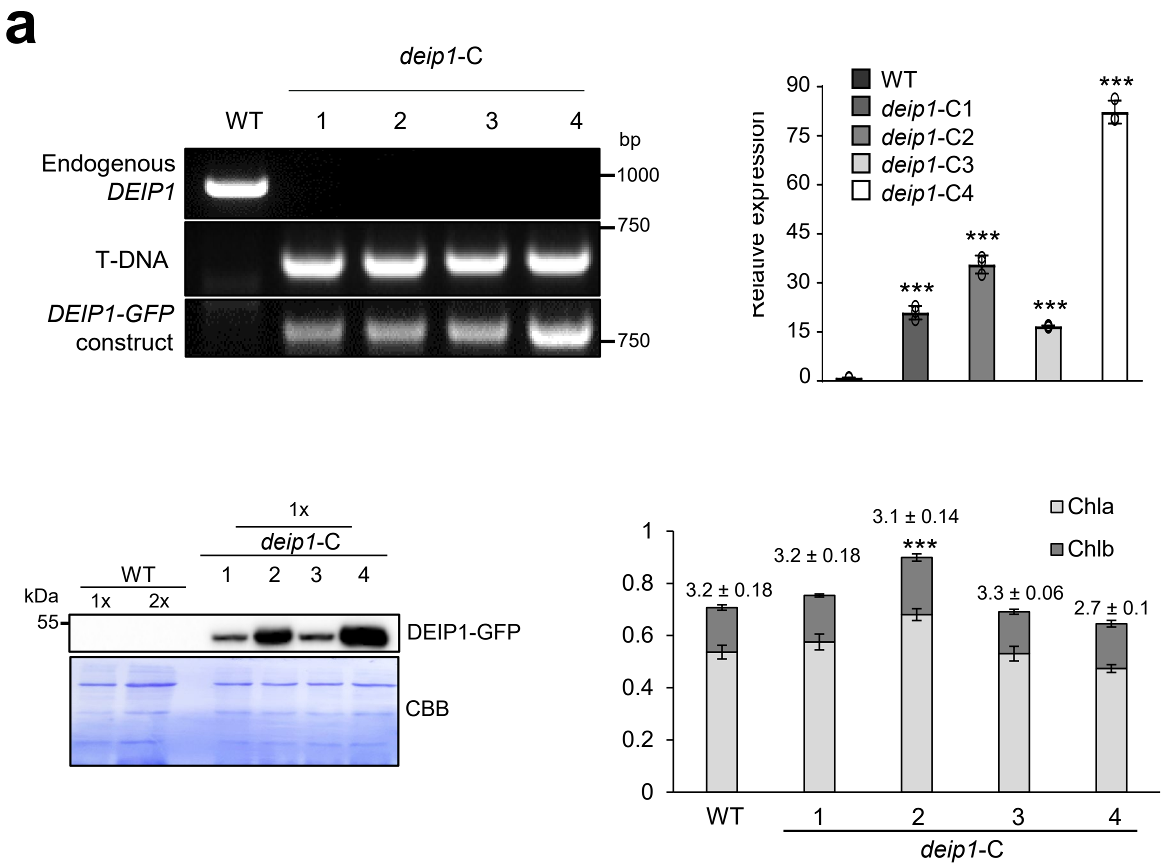

Supplementary Fig. 6. Complementation of the *deip1-1* mutant by introduction of a P_{UBQ10}::DEIP1-GFP expression construct. a Isolation of transgenic lines homozygous for both P_{UBQ10}::*DEIP1*-GFP and the *deip1-1* T-DNA insertion (*deip1*-C complemented lines). Shown are PCR assays for the presence of the endogenous *DEIP1* gene, the T-DNA insert, and the transgenically introduced DEIP1-GPF construct. **b** *DEIP1* transcript accumulation (relative to the wild type) in the complemented lines *deip1*-C1, 2, 3 and 4. n=3 independent biological replicates; error bars represent standard deviation; statistical significance was determined by one-sided ANOVA, p-values were adjusted by Tukey post-test for multiple comparisons and compared to the wild type, *** $P<0.001$. **c** Accumulation of the DEIP1-GFP fusion protein in the complemented lines 1, 2, 3 and 4. Samples of 10 µg total protein were loaded for the complemented lines (100%), and the blot was probed with an anti-GFP antibody. As a control for equal loading, the Coomassie-stained PAA gel (CBB) is shown below the immunoblot. **d** Chlorophyll contents in 7-day-old seedlings of wild-type (WT) plants and the four DEIP1-GFP complemented lines. The chlorophyll *a:b* ratio is given above each bar. n=3; error bars represent standard deviation; statistical significance was determined by one-sided ANOVA, p-values were adjusted by Tukey post-test for multiple comparisons and compared to the wild type; asterisks represent significant differences in the total chlorophyll content; *** $P<0.001$.

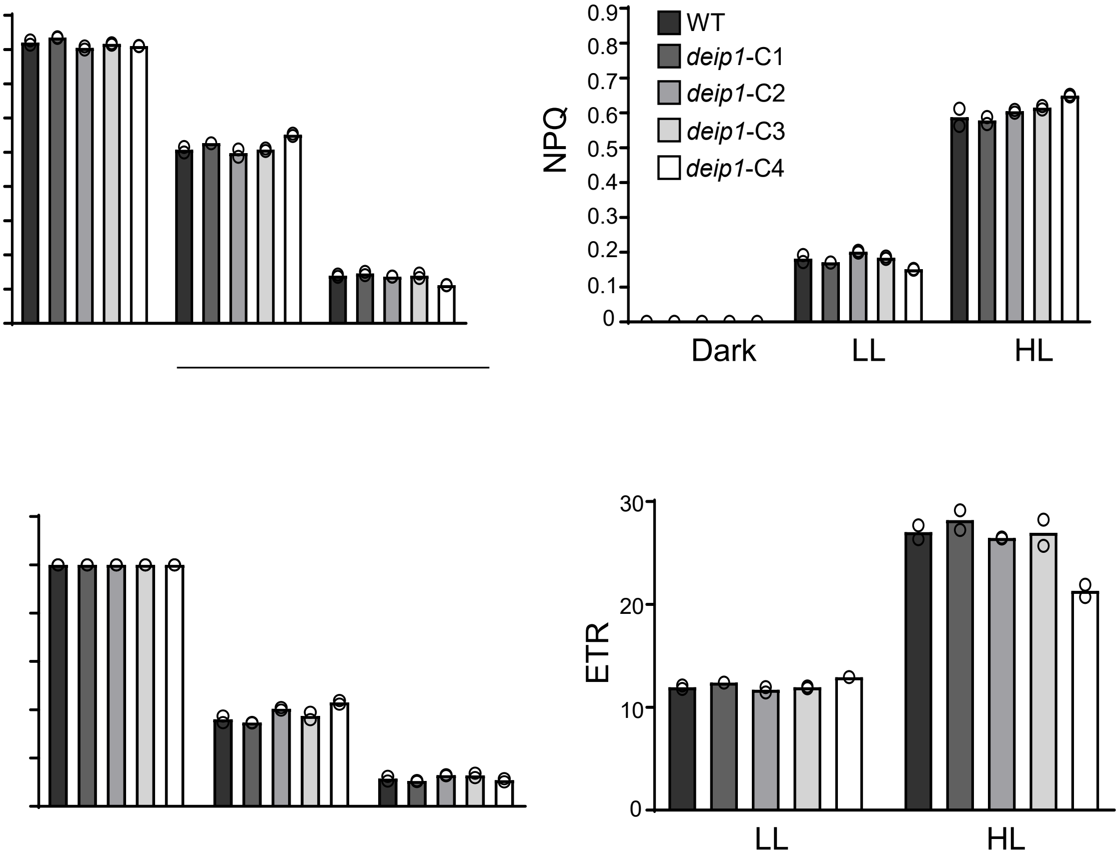

Supplementary Fig. 7. Measurement of photosynthetic parameters in wild-type plants and the P_{UBQ10}::DEIP1-GFP complemented lines (*deip1*-C). The parameters **a** maximum quantum yield (F_v/F_m) and effective quantum yield (ΦII), **b** non-photochemical quenching (NPQ), **c** open reaction centers of photosystem II (qL), and **d** electron transport rate (ETR) were obtained by PAM imaging of 7-day-old seedlings of the wild type (WT) and the four complemented lines *deip1*-C1-4. Each measurement was performed with an average of 8 seedlings. n=2 independent biological replicates. LL: low light (56 PAR); HL: high light (461 PAR).

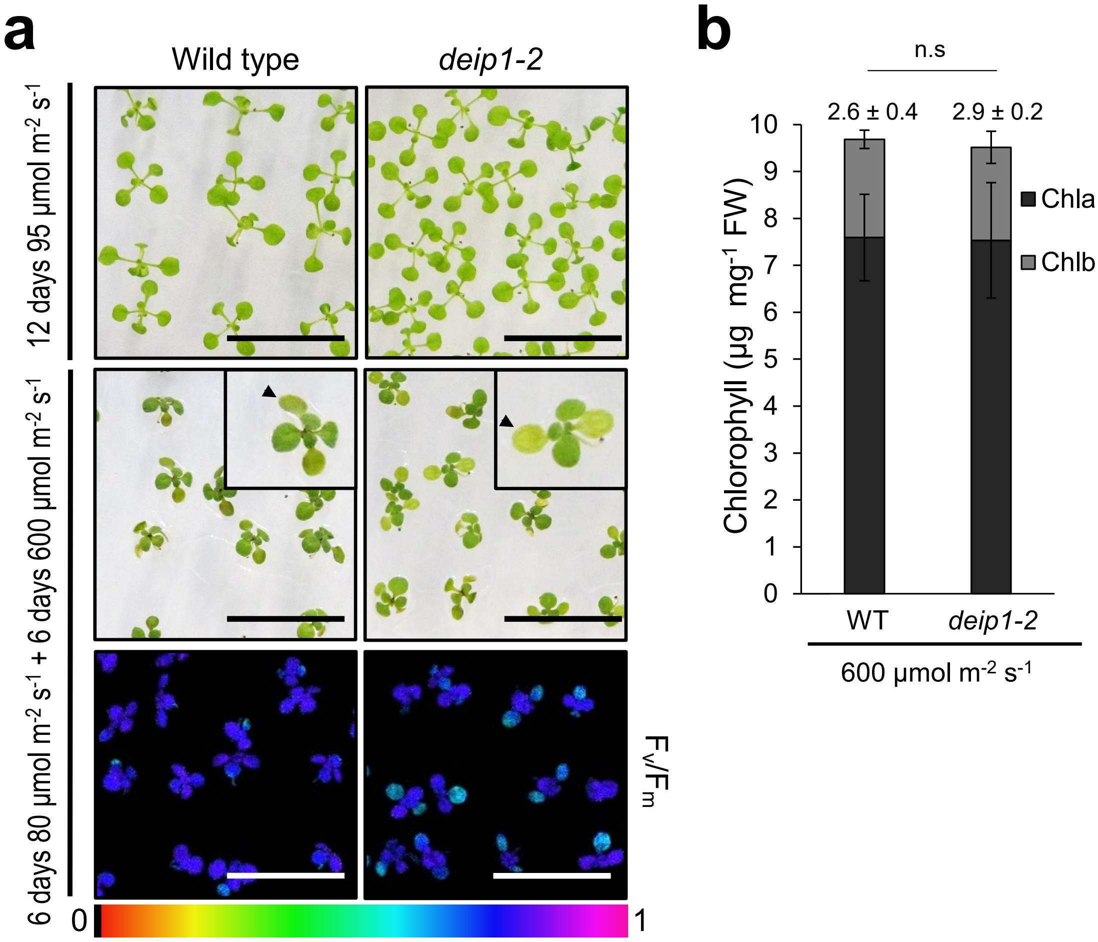

Supplementary Fig. 8. Analysis of wild-type and *deip1-2* seedlings grown under moderate light or high light. a Phenotypes and maximum quantum yield (F_v/F_m) of wild-type and *deip1-2* seedlings raised under moderate light intensity (95 µmol m^{-2} s^{-1}) for 12 days (upper panel), or grown under moderate light for 6 days and then transferred to high light (600 µmol m^{-2} s^{-1}) for 6 additional days (middle panel), followed by analysis by Imaging PAM (bottom panel). Arrowheads indicate pigmentation differences in the cotyledons of wild-type versus *deip1-2* seedlings. Scale bars: 2 cm; n=3 independent biological replicates. **b** Measurement of chlorophyll contents in wild-type (WT) and *deip1-2* seedlings at the end of the high-light treatment as shown in **a**. The chlorophyll *a* (dark grey) and chlorophyll *b* (light grey) contents were normalized to fresh weight, the chlorophyll *a:b* ratio is given above each bar. n=3; error bars represent standard deviation. Statistical significance was determined by paired two-tailed t-test; P = 0.173; n.s, not significantly different in total chlorophyll content.

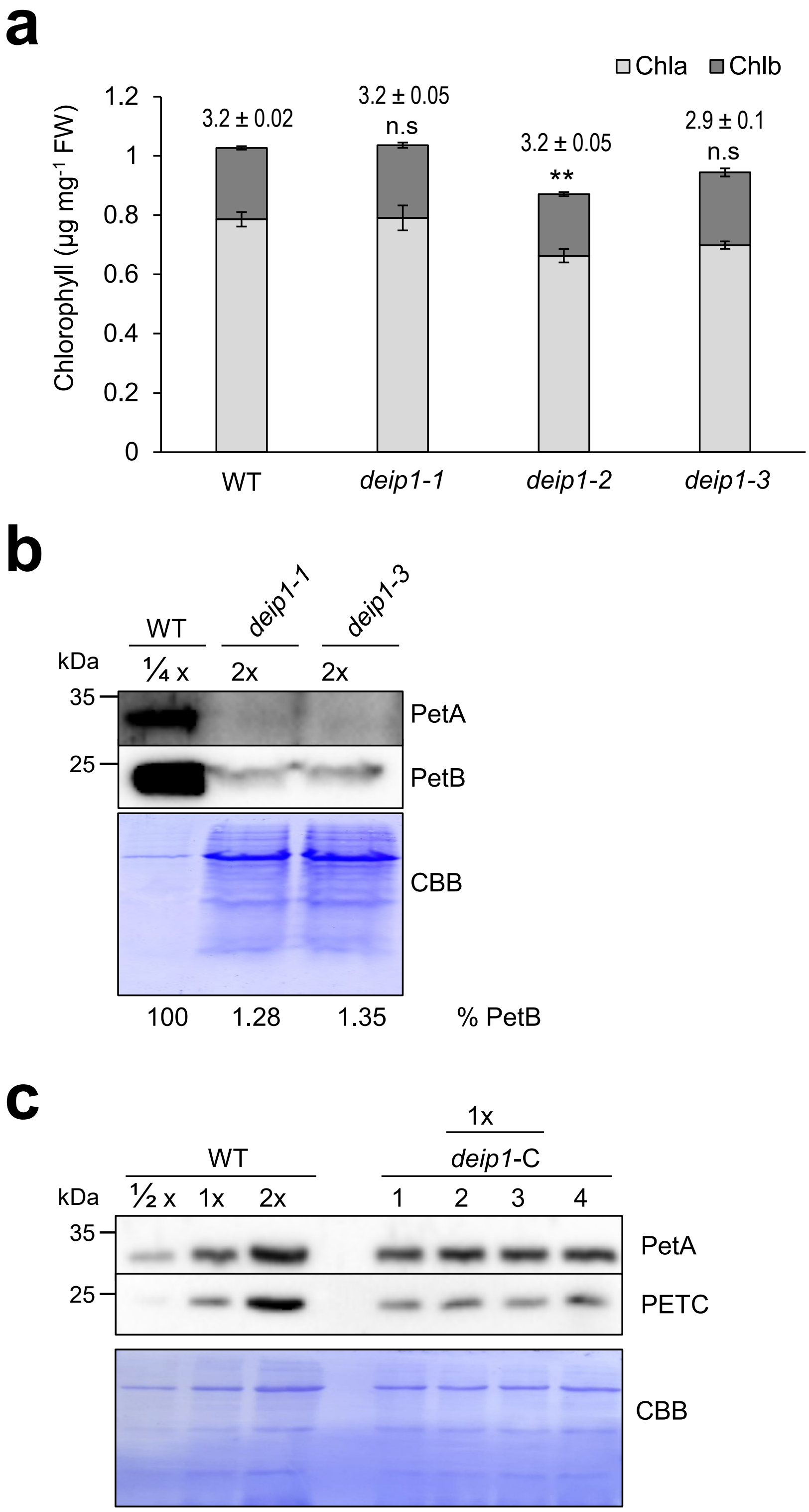

Supplementary Fig. 9. Chlorophyll contents of plants grown under low light, and accumulation of Cyt*b*$_6$*f* subunits in *deip1* mutants and *deip1*-C lines. a Chlorophyll contents of wild-type plants (WT), and *deip1-1*, *deip1-2* and *deip1-3* mutants grown for 5 weeks under low-light conditions. The chlorophyll *a* (light grey) and chlorophyll *b* (dark grey) contents were normalized to fresh weight, the chlorophyll *a:b* ratio is given above each bar. n=3; error bars represent standard deviation; statistical significance was determined by one-sided ANOVA, *P* values were adjusted by Tukey post-test and comparing total chlorophyll content to the wild type; ** *P*<0.01; ns, not significantly different in total chlorophyll content. **b** Accumulation of the Cyt*b*$_6$*f* subunits PetA and PetB in the wild type (WT) and the two *deip1*

knock-out mutants. Total protein samples of 2.5 µg for the wild type and 20 µg for the *deip1-1* and *deip1-3* mutants were electrophoretically separated in a 12.5% SDS-PAA gel. The Coomassie brilliant blue (CBB) staining is shown as loading control. n=3 independent biological replicates **c** Comparison of the accumulation of Cytb_6f subunits in the wild type (WT) and the *deip1*-C1-4 complemented lines. Samples of 10 µg total protein were loaded for the complemented lines (1x), and a dilution series of the wild-type protein (with 1x being equivalent to 10 µg total protein) was included to facilitate semiquantitative assessment. The blots were probed with antibodies against PetA and PETC, respectively. Coomassie brilliant blue (CBB) staining served as control for equal loading. n=2 independent biological replicates.

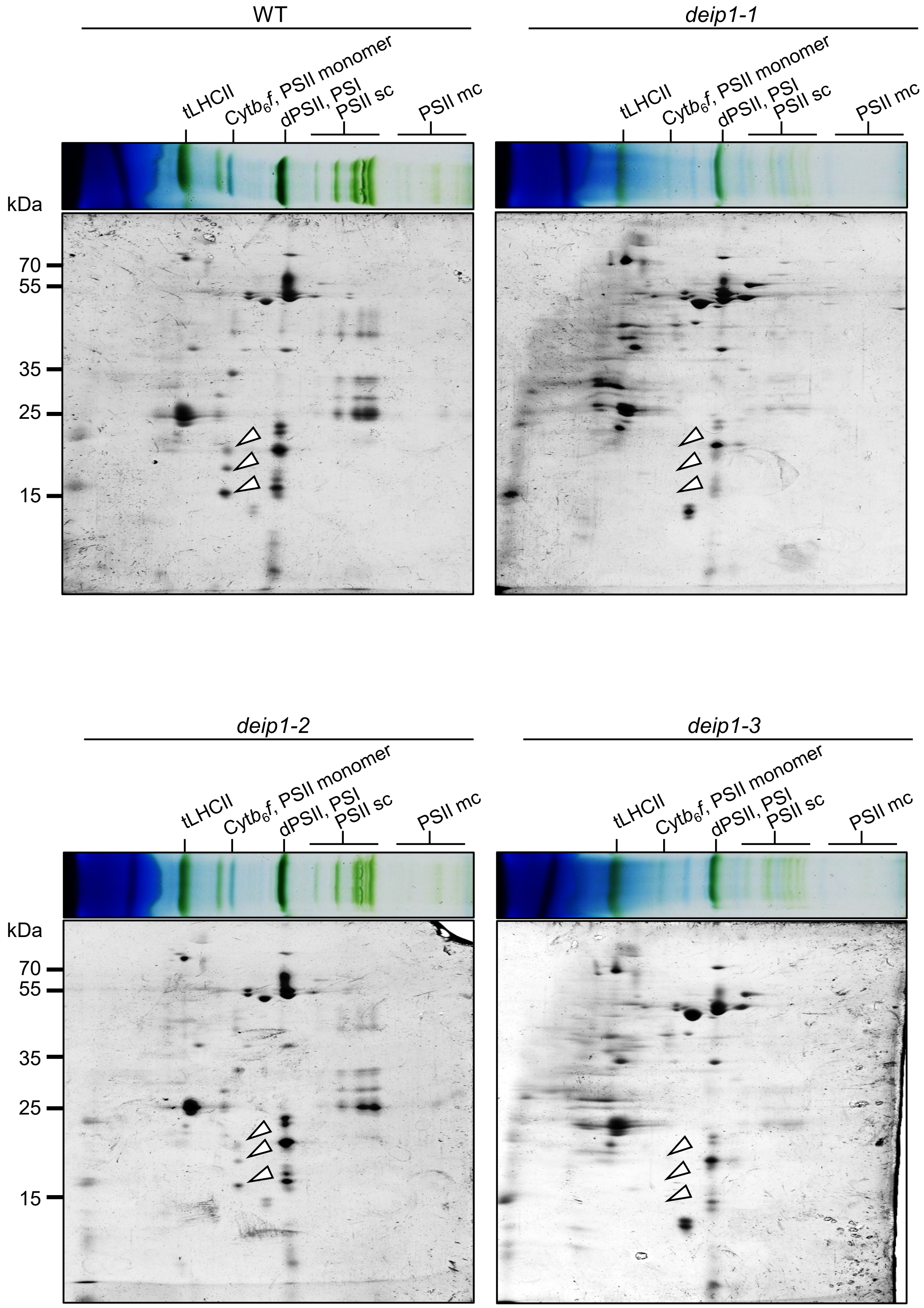

Supplementary Fig. 10. Analysis of thylakoids from wild-type plants and *deip1* mutants by two-dimensional SDS-polyacrylamide gel electrophoresis. The BN-PAGE in the first dimension was performed with thylakoid protein samples equivalent to 8 µg of chlorophyll. The

thylakoids were solubilized with β-DDM, and resolved in a 6-12.5% native gradient gel. The strips from the first-dimensional gel were solubilized with reducing sample buffer for SDS-PAGE for 1.5 h and placed on top of a 15% reducing SDS-PAA gel. After electrophoretic separation, the gel was fixed and the proteins were detected by silver staining. The positions of the LHCII trimer (tLHCII), Cytb_6f, PSII monomer, PSII dimer (dPSII), PSI, PSII supercomplexes (PSII sc) and PSII megacomplexes (PSII mc) are indicated at the top of the first dimensional BN-PAGE. White arrowheads indicate the Cytb_6f subunits present in the wild type and the *deip1-2* mutant, but absent from the *deip1-1* and *deip1-3* knock-out mutants. n= 3 independent biological replicates.

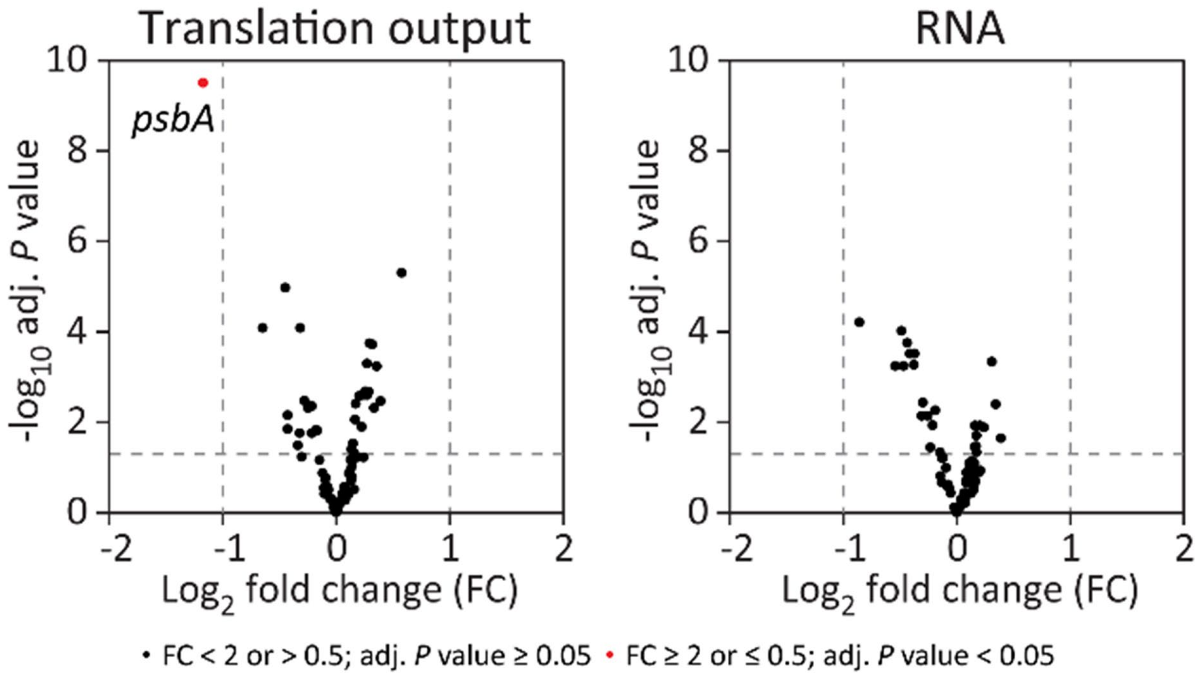

Supplementary Fig. 11. Volcano plots of translation output and mRNA abundances in the wild type and the *deip1-1* mutant. Changes in the translation output and transcript accumulation of chloroplast reading frames are shown in volcano plots. Log_2 values of fold change ratios of *deip1-1* versus wild type (x-axis) are plotted against the negative logarithm to the base of 10 of the adjusted *P* values (y-axis). Statistical significance was determined by the empirical Bayes method in the LIMMA package [80] and the *P* values were adjusted according to the False Discovery Rate (FDR) procedure [81]. Vertical and horizontal lines represent the cut-off for fold change (2-fold changes) and adjusted *P* values (adj. $P < 0.05$), respectively. *psbA* represents the only reading frame that shows a more than 2-fold change with statistical significance.

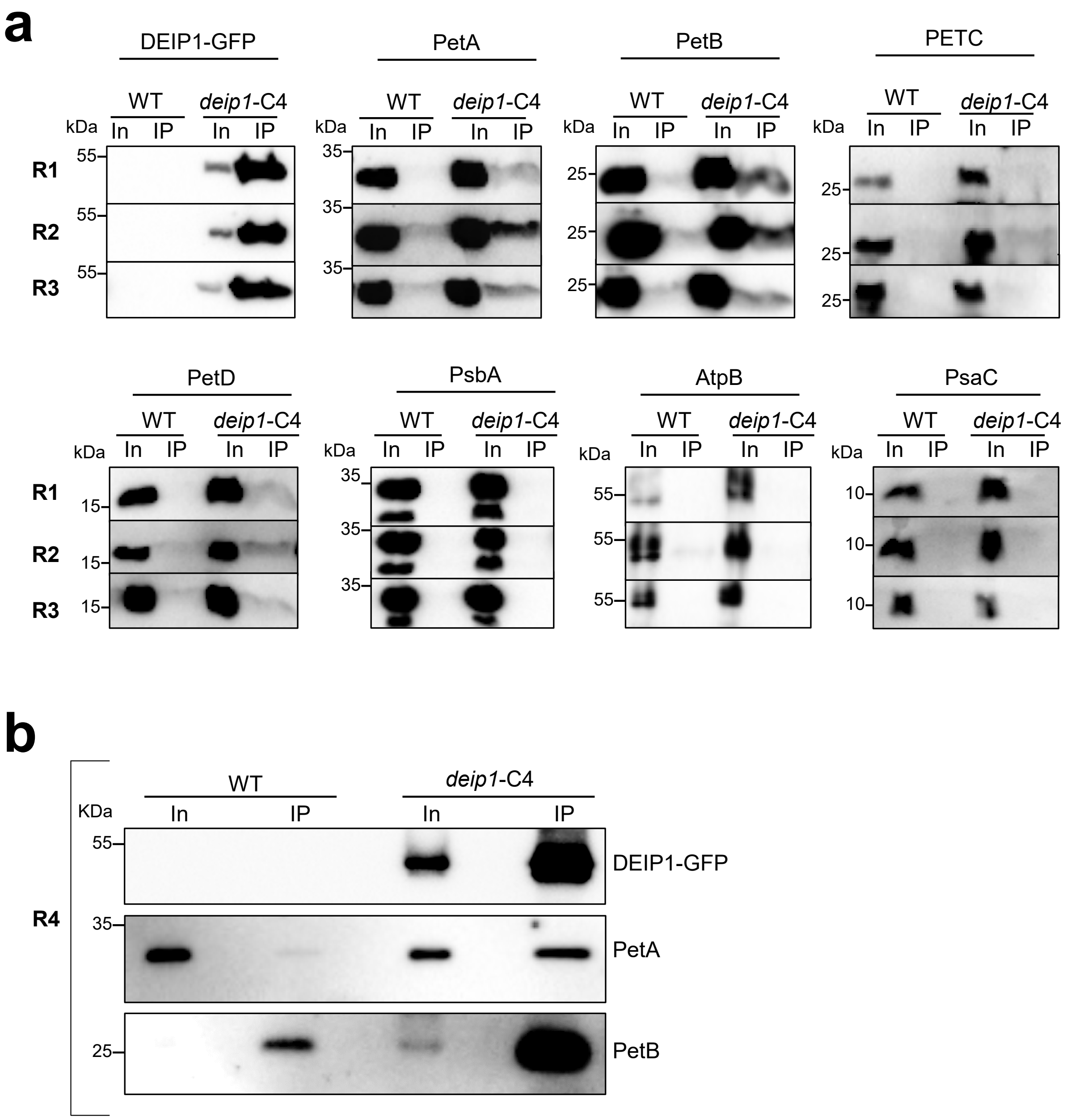

Supplementary Fig. 12. Co-IP in DSP crosslinked thylakoid samples from the wild type (WT) and the *deip1*-C4 complemented line. a Thylakoid samples were treated with 1.5 mM DSP as crosslinker prior to co-IP. The eluate was resolved by SDS-PAGE, and immunoblot detection was conducted with anti-GFP, anti-PetA, anti-PetB, anti-PETC, anti-PetD, anti-PsbA, anti-AtpB, and anti-PsaC antibodies. The biological replicates 1 (R1), 2 (R2) and 3 (R3) are indicated at the left. n= 3 independent biological replicates. **b** Additional biological replicate (R4) leaving an empty lane between each sample (to exclude the possibility that weakly hybridizing bands result from spillover between lanes). Immunoblot detection was conducted with anti-GFP, anti-PetA and anti-PetB antibodies. n= 1 biological replicate.

Supplementary Table 1. Entry name, source organism, amino acid sequence, gene name, protein name and length of the proteins used for phylogenetic tree construction and construction of an amino acid sequence alignment of putative DEIP1 orthologs.

Entry name (UniProtKB)	Organism	Amino acid sequence	Gene names	Protein names	Length (amino acids)
D8S030	*Selaginella moellendorffii*	MNKEKETKQSVETDAVNKDDGVEEVTRKFGLEAGLWKIFSSKKNESGEKKVQAKELLARYGGAYLVTSISLSAVSFALCYLLINAGVDVPSLLAKVGIQSNETGEKVGTLALAYAAHKAASPIRFPPTVALTPIVASWFGKKNEQEGGGKS	SELMODRAFT_105406	DUF1279 domain-containing protein	151
C5YG99	*Sorghum bicolor*	MAATALLLPSLAAAAPTPTQQLRLRLRRGSLACCSRSVRPPRRRLAVSAVQETKDGEAKTAEEITEKYGLEFGLWKVFSSKDDEEGGGERKKSRTDQAKELLAKYGGAYLATSISLSIVSFTLCYLLISAGVDVQDLLAKVGIVTGETGGKVGTFALAYAAHKAASPIRFPPTVALTPVVASWIGKIRKGGD	SORBI_3006G074500	DUF1279 domain-containing protein	192
A0A1D6EBF2	*Zea mays*	MAATALLLPSLAALTDTPRRAHRLLQPQLQLQRGSLVSRSLRPPRRRRLAVSAVQETKEGEAKTADEITEKYGLEFGLWKVFSSKEEEGGEGKKSRTDQAKELLAKYGGAYLATSISLSIVSFTLCYLLVSAGVDVQDLLAKVGIVTGETGGKVGTFALAYAAHKAASPIRFPPTVALTPVVASWIGKIRKGGD	ZEAMMB73_Zm00001d003756	Transmembrane protein C20orf108	194
A0A3B6AYM7	*Triticum aestivum*	MAAPALPLANHAAPTATARYLHRNHQLLQPKRQLPPGFLAARPLRQPRRPLSAVPETKEEDAKTAEEITEKYGLEVGLWKIFSSKEEEGGEGKPRKSRTEQAKELLGKYGGAYLATSITLSLISFTACYLLINAGVDVQQLLGKIGIATDETGGKVGTFALAYAAHKAASPIRFPPTVALTPVVANWIGKITKGGD	N.A*	DUF1279 domain-containing protein	196
A0A3B6DDA6	*Triticum aestivum*	MAAPALPLANHAAPTATARYLHRNLQLLQPKRQLPPGFLAARPLRQPRRALSAVPETKEEDAKTAEEITEKYGLEVGLWKIFSSKEEEGGEEGEEGKPKKSRTEQAKELLGKYGGAYLATSITLSLISFTACYLLINAGVDVQQLLGKIGIATDETGGKVGTFALAYAAHKAASPIRFPPTVALTPVVANWIGKITKGGD	N.A*	DUF1279 domain-containing protein	200
A0A3B6C756	*Triticum aestivum*	MAAPALPLANHAAPTATARYLHRNHQWLQPKRPGFLTARPLRQPRRALSAVPETKEEEAKTAEEITEKYGLEVGLWKIFSSKEEEGEEGKPKKSRTEQAKELLGKYGGAYLATSITLSLISFTACYLLINAGVDVQQLLGKIGIATDETGGKVGTFALAYAAHKAASPIRFPPTVALTPVVANWIGKITKGGD	N.A*	DUF1279 domain-containing protein	193
A0A287IFX2	*Hordeum vulgare* subsp. vulgare	RNIALHVAPRTYSMAQRWLLVTRFYLTFAFRGHRSQPSNPDSYLVPAPSFGGLRSASSAGSTPSNRPYRAPSMAAPALPLANHAAATATATARYLHRNHQLLQPKRQLPPGFLAARPLRQPRRPLSAVPETKEEEAKTADEITEKYGLEVGLWKIFSSKEEEEGGEEGKTKKSRTDQAKELLGKYGGAYLATSITLSLISFTACYLLINAGVDVQQLLGKIGIATDETGGKVGTFALAYAAHKAA	N.A*	DUF1279 domain-containing protein	274

		SPIRFPPTVALTPVVANWIGKIAKG GEGD			
I1IXK9	*Brachypodium distachyon*	MAAASALLLANLSAPTGTARYRHR LLQPRRQPPHQLLASRPLPLRQPL RPLSAVQDTKEETAKTAEEITEKYG LEVGLWKIFSSKEEEEGEGGKTKS RTDQAKELLAKYGGAYLATSITLSLI SFTACYLLINAGVDVQQLLTKVGIV TDETGGKVGTFALAYAAHKAASPI RFPPTVALTPVVANWIGKIRKGGD	100830285 BRADI_5g09630v3	DUF1279 domain-containing protein	196
Q7XTF4	*Oryza sativa* subsp. japonica	MAAGALLLANPAAPTARHQHRLLQ QRRQPRLLASSRPPRWRLSAVQE TKEGEAQTAEEITEKYGLEFGLWK VFSSKEGEEEEGKTRKSRTEQAKE LLAKYGGAYLATSITLSLISFTLCYL LVSAGVDVQDLLGKVGIATGETGG KVGTFALAYAAHKAASPIRFPPTVA LTPVVASWIGRIKKGGD	Os04g0416000 OSNPB_040416000	Os04g0416000 protein (cDNA, clone: J065144L23, full insert sequence)	187
A0A0K9PBH9	*Zostera marina*	MAIPAGTTATVTLRTAEIPIFFCSQR NLNTAIIVSLPKSRRRICAVQETKE PSTSSSSDSKLTEEETTEKYGLEV GLWKIFFTSKDGDSGGDGKGKTR KTDKAKDLLAKYGGAYLLTSISLSL VSFSLCYILVNAGIDVPGLLLKVGIS VDETGGKVGKLALAYAAHKAASPI RFPPTVALTPVVAGWIGKVKDGNT VTENENDIVE	ZOSMA_2G03320	DUF1279 domain-containing protein	208
D7TCP7	*Vitis vinifera*	MAGALLGTCFSSASLSINTREFRIG SSASTPPSKPNFTCFRVRAIKEKTE EIRTPSPSPSSSPSAQEITEKFGLE AGLWKIFSSKDEEKEGGKREKSKG EEAKELLAKYGGAYLATSITLSLISF SLCYALINAGVDVQALLQKVGISVD ATGEKVGTFALAYAAHKAASPIRFP PTVALTPIVASWIGKIVDKEN	VIT_06s0080g0110 0	DUF1279 domain-containing protein	196
A0A059AAP5	*Eucalyptus grandis*	MEAVNLFPALSPSSPRLRRDGYRF FAPSPRPCKPNSSRVAVRALREET KEARNPSPSSASADEVTKKYGLEA GLWQIFSSKEEQKGDDEKKSKTD QAKELLAKYGGAYLATSITLSLISFS LCYVLISAGIDVQALLQRVGISLDAT GEKVGTFALAYAAHKAASPIRFPPT VALTPIVAGWIGKKVDKEK	EUGRSUZ_J00538	DUF1279 domain-containing protein	191
A0A1U8HSP1	*Gossypium hirsutum*	MATTAVLTLTPLSSTSFLTAHTYSK LSSALTSTSIKPKFRSYQIKAIKEKA EEIQTPPSSSSSSSVDEVTKKYGLE VGLWKIFSSKEEGEQKKSKGDQAK ELLAKYGGAYLATSITLSLISFSLCY ALISAGVDVQALLQKVGISTDATGE KVGTFALAYAAHKAASPIRFPPTVA LTPIVAGWIGKKVEKDK	LOC107889195	uncharacterized protein LOC107889195	193
A0A1U8I0Y8	*Gossypium hirsutum*	MATTAVLTLTPLSSTSFLTAHTYSK LSSALTSTSIKPKFRSYQIKAIKEKA EEIQTPPSSSSSSSVDEVTKKYGLE VGLWKIFSSKEEGGQKKSKGDQA KELLAKYGGAYLATSITLSLISFSLC YALISAGVDVQALLQKVGISTDATG EKVGTFALAYAAHKAASPIRFPPTV ALTPIVAGWIGKKVEKDK	LOC107891572	uncharacterized protein LOC107891572	193
Q9XIN6	*Arabidopsis thaliana*	MAMMLLQIPPSSSLLNTRNLQIRFF HSSVSASSKKFRCRAVREKAEDID KNISPPSSSPPPPSAEEVTKKYGLE VGLWKILSSKDDEGSDGDNKKKKS KTDEAKELLAKYGGAYLATSITLSLI SFSLCYVLVTSGVDVQALLLKVGIS	At2g27290 F12K2.13 F12K2_13	At2g27290 (Expressed protein) (FAM210B-like protein, putative (DUF1279))	201

		TNETGEKVGAFALAYAAHKAASPI<br>RFPPTVALTPIVANWIGKKVDKEKD<br>DDK		(Uncharacterized<br>protein At2g27290)	
A0A078GVR3	*Brassica napus*	MAMILQVPSSTPSSLFHTRNTKTR<br>FFFFSSIQTSSESNTNKFRCRAVRE<br>KAEEKNTSPPSPEEVTKKYGLEVG<br>LWKILTSKDEESDGETKKKKKSKT<br>DEAKELLAKYGGAYLATSITLSLISF<br>SLCYALVTSGVDVQALLLKVGISTN<br>ETGEKVGAFALAYAAHKAASPIRFP<br>PTVALTPIVANWIGKKVDKEKDDE	BnaA04g15780D<br>DARMORV10_A04P<br>20870.1<br>GSBRNA2T000452<br>86001	(rape) hypothetical<br>protein<br>(BnaA04g15780D<br>protein)	197
M5WKS8	*Prunus persica*	MATAVLVPCLNSASFFNNNGQSR<br>NRCCASIQPSKASSYSKRFRVRAL<br>KEKTEEEIKNPSSADSAEEITKKYG<br>LEAGLWKIFSSKEEGKGGVENKSK<br>GDDAKQLLAKYGGAYLATSIILSIIS<br>FSLCYALVSAGIDVQALLQKVGISG<br>GETGEKVGTFALAYAAHKAASPIR<br>FPPTVALTPIVARWIGKKVEKEK	PRUPE_6G139600<br>PRUPE_ppa015327<br>mg	DUF1279 domain-<br>containing protein	194
B9HQ24	*Populus trichocarpa*	MAMATTTLQVQLPPFSSASFLNNK<br>SFKNYCCSTYIQPSNSSFKRLQTR<br>AIKEKTEEREAPSSSSSSSSSSSSV<br>EEVTKKYGLEAGLWKIFSSKEEEK<br>EEGEKTKSKGDQAKELLAKYGGAY<br>LATSITLSLISFSLCYALISAGIDVQA<br>VLLKVGISTDANGEKVGTFALAYAA<br>HKAASPIRFPPTVALTPIVAGWIGK<br>KADKEK	POPTR_009G16180<br>0	DUF1279 domain-<br>containing protein	204
B9SCZ5	*Ricinus communis*	MATPLQLSPPFPAASFLSNKNFCK<br>HYTGSLQPLKSTFKNFSTRAIKEKT<br>DSSSSSSSSAEDITKKYGLEAGLW<br>QIFSTKEERKEGDGETKKSKGDQA<br>KELLAKYGGAYLATSITLSFISFSLC<br>YALINAGIDVQALLQKVGISTDATG<br>EKVGTFALAYAAHKAASPIRFPPTV<br>ALTPIVATWIGKKVDKEKESP	RCOM_1282490	DUF1279 domain-<br>containing protein	194
I1LRK0	*Glycine max*	MTTSLLLPSPSCAAILSKGTNRICT<br>RASFHSLKSHVKKGFRVRALKEKT<br>EEIESPSQPSSPEEVTKKYGLEAGL<br>WQIFSSKEEGKDNSQQKSKGDQA<br>KELLAKYGGAYLATSITLSLISFALC<br>YALISAGIDVQALLQKVGISTDATG<br>EKVGTFALAYAAHKAASPIRFPPTV<br>ALTPIVAGWIGKKVEKDK	100811827<br>GLYMA_12G09330<br>0	DUF1279 domain-<br>containing protein	191
I1LKN2	*Glycine max*	MTTSLLLPSPSCASILSKGTNRICT<br>RASFHTLKSHVKKGFRVRALKEKT<br>EEIESPSQQSSPEEVTKKYGLEAG<br>LWQIFSSKEEGKDNSEQQKSKGD<br>QAKELLAKYGGAYLATSITLSLISFA<br>LCYALISAGIDVQSLLQKVGISTDAT<br>GEKVGTFALAYAAHKAASPIRFPPT<br>VALTPILAGWIGKKVEKDK	GLYMA_11G18020<br>0	DUF1279 domain-<br>containing protein	192
G7JH97	*Medicago truncatula*	MTTFSLLPSPSCASFLSNKKSSRF<br>CSLASIQSRKSNVKVLRVRAVKEK<br>TEEIKSSSKQSSPEEVTKKYGLEA<br>GLWKIFSSKEEGDQQKSKGDQAK<br>ELLAKYGGAYLATSITLSLISFALCY<br>VLINAGVDVQTLLQKVGISTDATGE<br>KVGTFALAYAAHKAASPIRFPPTVA<br>LTPIVAGWIGKKADKDK	11443444<br>MTR_4g052010<br>MtrunA17_Chr4g00<br>25891	FAM210B-like<br>protein	188
A0A0K9RQN9	*Spinacia oleracea*	MSRAASSFILSLSSSASSSSSIFHD<br>RTGSSRFIIYKKVTWLSSISNYRFQ<br>VRAVKDKTKDTPTPTPSSSSSSSS<br>SSADEITKKYGLEAGLWKIFSSKED<br>EGEEGKKEKTKGEEAKELLTKYGG<br>AYLATSITLSVISFTICYALINAGVDV	SOVF_045480	DUF1279 domain-<br>containing protein	208

		SALLQKLGISSTETGEKVGTFALAYAAHKAASPIRFPPTVALTPIVASWIGKKVDKDN			
A0A2G2YH74	*Capsicum annuum*	MMASALQVCSQYYYNNNRIAVCSLFTVFPNLRKPNKVLNSATSNSNNKFKVRALKEKTTEEEVKSAEEITKKFGLEAGLWKIFSSKDEGDEENKDKKSKGDQAKELLAKYGGAYLATSITLSVISFALCYVLINAGVDVQALLQKVGISTDATGEKVGTFALAYAAHKAASPIRFPPTVALTPIVASWIGKKVDKDE	T459_28564	DUF1279 domain-containing protein	197
A0A3Q7IZZ8	*Solanum lycopersicum*	MASALQISSQYSKIALCSLFPVFPNLRKPNKALNSVTSNSNKKFKIRALKEKTTEEVKSAEEITKKFGLEAGLWKIFSSKEDRDEENKDKKSKGDQAKELLAKYGGAYLATSITLSLISFGLCYALINSGVDVQSLLQKVGISTDETGEKVGTFALAYAAHKAASPIRFPPTVALTPIVATWIGKKVDKEK	101243761	DUF1279 domain-containing protein	191
M1BQD2	*Solanum tuberosum*	MASALQISSQHSKIALCSLFPVFPNLRKPNKVLNSGTSNSNKKFKIRALKEKTTEEVKSAEEITKKFGLEAGLWKIFSSKEDGDEENKDKKSKGDQAKELLAKYGGAYLATSITLSLISFGLCYALINSGVDVQSLLQKVGISTDETGEKVGTFALAYAAHKAASPIRFPPTVALTPIVASWIGKKVDKEK	102594292	OJ991214_12.13 protein	191
A0A1S4B0Q1	*Nicotiana tabacum*	MASAWQICSQYYHNRIALCSISPTLPIFRKPNRVLNSATSNTKIKFKVRALKEKTEEINSAEEITKKYGLEVGLWKIFSSKEEGEEENKEKKSKGDQAKELLAKYGGAYLATSITLSLISFTLCYALINAGVDVQSLLQKVGISTDETGEKVGTFALAYAAHKAASPIRFPPTVALTPIVASWIGKKADKE	LOC107803330	uncharacterized protein LOC107803330	191
A0A1S4CIL3	*Nicotiana tabacum*	MASASQISSHYYHNRIALCSVSPALPILSKPNRVLNSTTSNSKNKFKVKALKEKTEEIKSAEEITKKYGLEVGLWKIFSSKEEGEEENKEKKSKGDQAKELLGKYGGAYLATSITLSLISFTLCYALINAGVDVQSLLQKVGISTDETGEKVGTFALAYAAHKAASPIRFPPTVALTPIVASWIGKKADKEK	LOC107819445	uncharacterized protein LOC107819445	192
A0A2J6LGM0	*Lactuca sativa*	MGAIHFAAPSASACITNKEFNIILCSGNGTKRRVKSFMTTSSSCSSGSRRRSGSVVIRAIKEETKPKQSDSSSSPDVITQKYGLEAGLWKIFSSKEEEEDENVDVKKSKGDQAKELLTKYGGAYLATSITLSLISFSLCYALITAGVDVQALLQKVGISANETGEKMGTFALAYAAHKAASPIRFPPTVALTPIVASWIGKKVDKDN	LSAT_2X35940	DUF1279 domain-containing protein	207
A0A251SEL7	*Helianthus annuus*	MAMAAIPAAAASVYNEFNGTRINAVFSNFKPTTRTSVKIRAIKEETQQRQTNTSSSSSPDEITKKYGLEVGLWKIFSSKEDEEGDGKKSKGDQAKELLTKYGGAYLATSITLSLISFSLCYALITAGVDVPALLQKVGISAGETGEKVGTFALAYAAHKAASPIRFPPTVALTPIVASWIGKKVDKDN	HannXRQ_Chr14g0433141	DUF1279 domain-containing protein	188
U5D740	*Amborella trichopoda*	MASAMALSPLPASLHLSKTFLHNSNSLYFKSLPASPLSNGTKRLFISRALQETKEKDREGAQSAEQITQKFGLEAGLWKIFGSKEAKEGEGKSKANQTKELLAKYGGAYLATSIFLSLVSFALCYLLVRAGIDIQGLLEKVGIHADET	AMTR_s00030p00231690	DUF1279 domain-containing protein	192

		GGKVGTFALAYAAHKAASPIRFPPTVALTPMVANWIRKKAKEDK			
A0A2K1JIB9	*Physcomitrium patens*	MSLLSTDLVHEWRTSLPVHASPVVSSNVRPSSCGFRSKLKGGKVGPLSTMPVLGLVGSKRSGVLCAATKEGKEKEQDTKDIDAETVTKKYGLEAGLWKIFSSKDKENSPEQGTKTNQAKELLKRYGGAYLVTSISLSIVSFSLCYVLVQAGVDVTSLLDKVGIHANDTGEKVGTFALAYAAHKALSPVRFPPTVALTPIVAGWFGKKPEDDNDKTCS	PHYPA_018704	DUF1279 domain-containing protein	217
Q00VU7	*Ostreococcus tauri*	MARSGARRETRASARARASCARAWTVNDARRARWSTRARARTERERERVEEVTKKWGLEAGLWRVWKSDAKAEGGGDGENPVGESLSRMDMAKNLLKRYGSAYLATSISLSLISITVFYFLVAGGIDVAALLEKIGITVNATSEQFGTFALAYAAHKASSPIRFGPTVALTPLVARWMGKDVDDAEERTTDETDGGAP	OT_ostta14g02235	DUF1279 domain-containing protein	197
A8IL11	*Chlamydomonas reinhardtii*	MQSTVQLTRRSTVVRRCEGRICRPFAPCHPVSHIAKASPATEEPKTANKSEAELATEKFGLEAGLLTALTSKDEGGEGKLSNTEQAKRLLAQYGSAYLITSISFAIVSFAACYLAVDSGVDMAGVLARFGLEASDTSEKVGTFALAYAAHKALSPVRFPPTVALTPVVAKYLGKKKEEPSSGSNSK	CHLRE_12g485850v5 CHLREDRAFT_183068	Predicted protein	186
A0A1U7YJF7	Nicotiana silvestris	MASAWQICSQYYHNRIALCSISPTLPIFRKPNRVLNSATSNTKIKFKVRALKEKTEEINSAEEITKKYGLEVGLWKIFSSKEEGEEENKEKKSKGDQAKELLAKYGGAYLATSITLSLISFTLCYALINAGVDVQSLLQKVGISTDETGEKVGTFALAYAAHKAASPIRFPPTVALTPIVASWIGKKADKE	LOC104247735	uncharacterized protein LOC104247735	191

* not annotated (N.A)

Supplementary Table 2. List of synthetic oligonucleotides used in this study.

Primer Name	Primer sequence (5' to 3')	Function
cl_AT2G27290_For	AACAGGTCTCAGGCTCGATGGCGATGATGCTTCTGC	Genotyping and cloning of the gene AT2G27290 (forward primer). The sequence in red is derived from the CDS of the target gene. The sequence in black corresponds to the adaptors for vector pGGC000.
cl_AT2G27290_Rev	AACAGGTCTCACTGACTTGTCATCATCCTTCTCCTTG	Genotyping and cloning of the gen AT2G27290 (reverse primer). The sequence is derived from the CDS of the target gene. The sequence in black corresponds to the adaptors for vector pGGC000.
gt_AT2G27290-Intron_Rev	GCTTACATAACTTCTACAACTACCACG	Genotyping of the SALK_037263 T-DNA insertion.
cc_AT2G27290_sgRNA1_For (Rev_Comp)	ACCAGGTCTCaATTGGTGGAAGAATCGGATTTGAGTTTTAGAGCTAGAAATAGCAAG	Generation of CRISPR/Cas9 lines. The sequence in red corresponds to sgRNA1.
cc_AT2G27290_sgRNA1_Rev_Comp	TGGTGGTCTCTAAACCTTTCTTCCCAATCCAATTCAATCTCTTAGTCGACTCTACC	Generation of CRISPR/Cas9 lines. The sequence in red corresponds to the reverse complemented sgRNA2.
qAT2G27290_For	GAGACAGGAGAGAAAGTAGGAG	Amplification of AT2G27290 by qRT-PCR (forward primer)
qAT2G27290_Rev	CCACTTTCTTCCCAATCCAATT	Amplification of AT2G27290 by qRT-PCR (reverse primer)
qPORA-For	GACGAAGTCGGGAGTGTA	Amplification of *PORA* (protochlorophyllide oxidoreductase A, AT5G54190) by qRT-PCR (forward primer)
qPORA-Rev	CTTCTCGCTGACTTCCCAAA	Amplification of PORA (protochlorophyllide oxidoreductase A, AT5G54190) by qRT-PCR (reverse primer)
qLHCB2_For	ATGGGCTATGTTGGGTGCTC	Amplification of *LHCB2.1* (light-harvesting chlorophyll B 2.1, AT2G05100) by qRT-PCR (forward primer)
qLHCB2_Rev	CCACCTCCGATTCTGTAGCC	Amplification of *LHCB2.1* (light-harvesting chlorophyll B 2.1 ,AT2G05100) by qRT-PCR (reverse primer)
qUBQ10_For	GGCCTTGTATAATCCCTGATGAATAAG	Amplification of *UBQ10* (ubiquitin 10, AT4G05320) by qRT-PCR (forward primer)
qUBQ10_Rev	AAAGAGATAACAGGAACGGAAACATAGT	Amplification of *UBQ10* (ubiquitin 10, AT4G05320) by qRT-PCR (reverse primer)
DEIP1-p1p4-For	GGGGACAAGTTTGTACAAAAAAGCAGGCTTAATGGCGATGATGCTTCTGC	Amplification of the CDS of *DEIP1* for cloning into the pDONR™221 P1P4 plasmid for BIFC assays (forward primer)
DEIP1-p1p4-Rev	GGGGACAACTTTGTATAGAAAAGTTGGGTGCTTGTCATCATCCTTCTCCT	Amplification of the CDS of *DEIP1* for cloning into the pDONR™221 P1P4 plasmid for BIFC assays (reverse primer)
DEIP1-p3p2-For	GGGGACAACTTTGTATAATAAAGTTGGAATGGCGATGATGCTTCTGC	Amplification of the CDS of *DEIP1* for cloning into the pDONR™221 P3P2 plasmid for BIFC assays (forward primer)
DEIP1-p3p2-Rev	GGGGACCACTTTGTACAAGAAAGCTGGGTGCTTGTCATCATCCTTCTCCT	Amplification of the CDS of *DEIP1* for cloning into the pDONR™221 P3P2 plasmid for BIFC assays (reverse primer)
RBCS-ctp-p3p2-For	GGGGACAACTTTGTATAATAAAGTTGGAATGGCTTCTATGATATCCTC	Amplification of the sequence encoding the pea RBCS chloroplast transit peptide for cloning into the pDONR™221 P3P2 plasmid for BIFC assays (forward primer)
RBCS-PetA-Rev	CAAGAAAAGGTATTTCTAGTTTGTTCGGAATCGGTAAGGTCAG	Amplification of the sequence encoding the pea RBCS chloroplast transit peptide, with overhanging sequence for amplification of *petA* (reverse primer)

RBCS- PetA-For	CTGACCTTACCGATTCCGAACAAACTAGAAATACCTTTTCTTG	Amplification of the CDS of *petA,* with overhang for the sequence encoding the pea RBCS chloroplast transit peptide (forward primer)
PetA-p3p2-Rev	GGGGACCACTTTGTACAAGAAAGCTGGGTGAAAATTCATTTCGGATAATTGAACC	Amplification of the CDS of *petA* for cloning into the pDONR™221 P3P2 plasmid for BIFC assays (reverse primer)
RBCS-PetB-Rev	CTTCGAACCAATCATAAACTTTACTTCTAGACATGCACTTTACTCTTC	Amplification of the sequence encoding the pea RBCS chloroplast transit peptide, with overhang for amplification of *petB* (reverse primer)
RBCS-PetB-For	GAAGAGTAAAGTGCATGTCTAGAAGTAAAGTTTATGATTGGTTCGAAG	Amplification of the CDS of *petB,* with overhang for the sequence encoding the pea RBCS chloroplast transit peptide (forward primer)
PetB-p3p2-Rev	GGGGACCACTTTGTACAAGAAAGCTGGGTGTAAGGGACCAGAAATACCTTG	Amplification of the CDS of *petB* for cloning into the pDONR™221 P3P2 plasmid for BIFC assays (reverse primer)
RBCS-PETC-Rev	CTTGACATCGCCATCAAAGCATCTAGACATGCACTTTACTCTTC	Amplification of the sequence encoding the pea RBCS chloroplast transit peptide, with overhang for amplification of *PETC* (reverse primer)
RBCS- PETC-For	GAAGAGTAAAGTGCATGTCTAGATGCTTTGATGGCGATGTCAAG	Amplification of the CDS of *PETC*, with overhang for the sequence encoding the pea RBCS chloroplast transit peptide (forward primer)
PETC-p3p2-Rev	GGGGACCACTTTGTACAAGAAAGCTGGGTGAGACCACCATGGAGCATC	Amplification of the CDS of *PETC* for cloning into the pDONR™221 P3P2 plasmid for BIFC assays (reverse primer)
RBCS-PetD-Rev	CAAATCTGGTTTTTTTGTTACTCCTCTAGACATGCACTTTACTCTTC	Amplification of the sequence encoding the pea RBCS chloroplast transit peptide, with overhang for amplification of *petD* (reverse primer)
RBCS-PetD-For	GAAGAGTAAAGTGCATGTCTAGAGGAGTAACAAAAAAACCAGATTTG	Amplification of the CDS of *petD*, with overhang for the sequence encoding the pea RBCS chloroplast transit peptide (forward primer)
PetD-p3p2-Rev	GGGGACCACTTTGTACAAGAAAGCTGGGTGAAAAAGACCTAAAGTTAGAGATTTATC	Amplification of the CDS of *petD* for cloning into the pDONR™221 P3P2 plasmid for BIFC assays (reverse primer)

Supplementary Table 3. Antibodies used in this study.

Antibody	Supplier	Cat.No / Ref.	Antibody dilution
anti-GFP	TaKaRa (ClonTech) Living Colors A.v. Monoclonal Antibody (JL-8)	632381	1:1000
anti-PsaB	Agrisera	AS10 695	1:1000
anti-PsaC	Agrisera	AS10 939	1:1000
anti-PSAD	Agrisera	AS09 461	1:1000
anti-LHCA1	Agrisera	AS01 006	1:2000
anti-PsbA	Agrisera	AS10 704	1:2500
anti-PsbD	Agrisera	AS06 146	1:2500
anti-PSBO	Agrisera	AS06 142-33	1:000
anti-LCHB2	Agrisera	AS01 003	1:1000
anti-PetA	Agrisera	AS06 119	1:1000
anti-PetB	Agrisera	AS18 4169	1:1000
anti-PETC	Agrisera	AS08 330	1:1000
anti-PetD	provided by Dr. Stephan Greiner	Schwenkert et.al., 2007 [26]	1:1000
anti-AtpB	Agrisera	AS05 085	1:5000
anti-AtpE	Agrisera	AS10 1586	1:1000
anti-RbcL	Agrisera	AS03 037	1:5000
anti-CURT1A	Agrisera	AS08 316	1:1000
