# Supplementary material for: De-etiolation-induced protein 1 (DEIP1) mediates assembly of the cytochrome b6f complex in Arabidopsis
Source: Nat Commun. 2022 Jul 13;13:4045. doi: 10.1038/s41467-022-31758-7 (PMC9279372; doi:10.1038/s41467-022-31758-7)
Supplement: Supplementary file 3 — Description of Additional Supplementary Files [file 41467_2022_31758_MOESM3_ESM.pdf]

Description of Additional Supplementary Files

File Name: Supplementary Data 1
Description: Ribosome profiling of plastid-encoded genes. Data from comparative transcript and ribosome profiling analyses of wild-type plants and deip1-1 mutant plants are shown.
