# Supplementary material for: De-etiolation-induced protein 1 (DEIP1) mediates assembly of the cytochrome b6f complex in Arabidopsis
Source: Nat Commun. 2022 Jul 13;13:4045. doi: 10.1038/s41467-022-31758-7 (PMC9279372; doi:10.1038/s41467-022-31758-7)
Supplement: Supplementary file 5 — Reporting Summary [file 41467_2022_31758_MOESM5_ESM.pdf]

nature portfolio

Corresponding author(s): Ralph Bock

Last updated by author(s): Jun 16, 2022

Reporting Summary

Nature Portfolio wishes to improve the reproducibility of the work that we publish. This form provides structure for consistency and transparency in reporting. For further information on Nature Portfolio policies, see our Editorial Policies and the Editorial Policy Checklist.

Statistics

For all statistical analyses, confirm that the following items are present in the figure legend, table legend, main text, or Methods section.

n/a	Confirmed	
☐	☒	The exact sample size (*n*) for each experimental group/condition, given as a discrete number and unit of measurement
☐	☒	A statement on whether measurements were taken from distinct samples or whether the same sample was measured repeatedly
☐	☒	The statistical test(s) used AND whether they are one- or two-sided<br>*Only common tests should be described solely by name; describe more complex techniques in the Methods section.*
☐	☒	A description of all covariates tested
☐	☒	A description of any assumptions or corrections, such as tests of normality and adjustment for multiple comparisons
☐	☒	A full description of the statistical parameters including central tendency (e.g. means) or other basic estimates (e.g. regression coefficient) AND variation (e.g. standard deviation) or associated estimates of uncertainty (e.g. confidence intervals)
☐	☒	For null hypothesis testing, the test statistic (e.g. F, t, r) with confidence intervals, effect sizes, degrees of freedom and P value noted<br>*Give P values as exact values whenever suitable.*
☒	☐	For Bayesian analysis, information on the choice of priors and Markov chain Monte Carlo settings
☒	☐	For hierarchical and complex designs, identification of the appropriate level for tests and full reporting of outcomes
☐	☒	Estimates of effect sizes (e.g. Cohen's *d*, Pearson's *r*), indicating how they were calculated

Our web collection on statistics for biologists contains articles on many of the points above.

Software and code

Policy information about availability of computer code

Data collection	For microarray, raw data extraction was done with GenePix Pro 7.2.29.002, all further data analyses were done with Microsoft Excel. Protein information (gene ID and sequences) was retrieved from UniProt.
Data analysis	For microarray data, statistical analysis was done in R. For construction of phylogenetic trees and protein sequence alignments, the online software http://www.phylogeny.fr/index.cgi was employed.

For manuscripts utilizing custom algorithms or software that are central to the research but not yet described in published literature, software must be made available to editors and reviewers. We strongly encourage code deposition in a community repository (e.g. GitHub). See the Nature Portfolio guidelines for submitting code & software for further information.

Data

Policy information about availability of data

All manuscripts must include a data availability statement. This statement should provide the following information, where applicable:

- Accession codes, unique identifiers, or web links for publicly available datasets
- A description of any restrictions on data availability
- For clinical datasets or third party data, please ensure that the statement adheres to our policy

The data supporting the findings of this study are available within the paper and its supplementary information files. Genetic material and all raw data are available upon request from the corresponding author.

Field-specific reporting

Please select the one below that is the best fit for your research. If you are not sure, read the appropriate sections before making your selection.

☒ Life sciences ☐ Behavioural & social sciences ☐ Ecological, evolutionary & environmental sciences

For a reference copy of the document with all sections, see nature.com/documents/nr-reporting-summary-flat.pdf

Life sciences study design

All studies must disclose on these points even when the disclosure is negative.

Sample size	To achive robustness in the data, we used pools of at least 50-150 seedlings or 6-8 plants per each biological replicate. Each biological replicate represents an independent experiment. Variation in the data was analyzed by statistical methods (Pearson correlation or Tukey post test). Appropriate controls were included to verify that our experimental procedures were consistent with previous experimental findings.
Data exclusions	No data were excluded from the analyses.
Replication	Statistical analyses (including Pearson correlation and Tukey post test) were performed for all quantitative data. For qualitative and semiquantitative data (e.g., protein analyses), the experiments were conducted with 2 to 5 biological replicates, and at least two technical replicates.
Randomization	Plant growth and harvest of material was randomized to exclude positional effects during growth or harvesting time.
Blinding	Due to the technical procedures employed, blinding was not applicable.

Reporting for specific materials, systems and methods

We require information from authors about some types of materials, experimental systems and methods used in many studies. Here, indicate whether each material, system or method listed is relevant to your study. If you are not sure if a list item applies to your research, read the appropriate section before selecting a response.

Materials & experimental systems

n/a	Involved in the study	
☐	☒	Antibodies
☒	☐	Eukaryotic cell lines
☒	☐	Palaeontology and archaeology
☐	☒	Animals and other organisms
☒	☐	Human research participants
☒	☐	Clinical data
☒	☐	Dual use research of concern

Methods

n/a	Involved in the study	
☒	☐	ChIP-seq
☒	☐	Flow cytometry
☒	☐	MRI-based neuroimaging

Antibodies

Antibodies used	Commercially available antibodies were employed using the dilutions recommended by the manufacture. Antibodies are listed below, including name, supplier and catalogue number (Cat. #):<br>anti-GFP, TaKaRa (ClonTech) Living Colors A.v. Monoclonal Antibody (JL-8), Cat.# 632381; anti-PsaB, Agrisera, Cat# AS10 695; anti-PsaC, Agrisera, Cat. #AS10 939, anti-PsaD, Agrisera, Cat. # AS09 461; anti-LHCB1, Agrisera, Cat. # AS01 005; anti-PsbA, Agrisera, Cat. # AS10 704, anti-PsbD, Agrisera, Cat. # AS06 146, anti-PSBO, Agrisera, Cat. # AS06 142-33; anti-LCHB2, Agrisera, Cat. # AS01 003; anti-PetA, Agrisera, Cat. # AS06 119, anti-PetB, Agrisera, Cat. # AS18 4169; anti-PETC, Agrisera, Cat. # AS08 330; anti-AtpB, Agrisera, Cat. # AS05 085, anti-AtpE, Agrisera, Cat. # AS10 1586; anti-RbcL, Agrisera, Cat. # AS03 037; anti-CURT1A, Agrisera, Cat. # AS08 316.<br>The antibody anti-PetD was kindly provided by Dr. Stephan Greiner, and is described in Schwenkert et.al., 2007; doi: 10.1104/pp.107.100131. The anti-PetD antibody was used at a dilution of 1:1000.
Validation	All commercially available antibodies were validated by the supplier. The antibody anti-PetD was validated in Schwenkert et.al., 2007; doi: 10.1104/pp.107.100131.

Animals and other organisms

Policy information about studies involving animals; ARRIVE guidelines recommended for reporting animal research

Laboratory animals	The study did not involve laboratory animals.

Wild animals	The study did not involve wild animals.
Field-collected samples	The study did not involve samples collected from field.
Ethics oversight	No ethical approval or guidance was required for plant material.

Note that full information on the approval of the study protocol must also be provided in the manuscript.
